# Enhancing Hybrid Photovoltaic–Thermal System Efficiency with Boron Dipyrromethene Dyes

**DOI:** 10.1021/acsaom.4c00309

**Published:** 2024-09-04

**Authors:** Kenneth Coldrick, Craig Newman, John Doran, George Amarandei, Mikhail A. Filatov

**Affiliations:** † School of Physics, Clinical and Optometric Sciences, Technological University Dublin, City Campus, Grangegorman Lower, D07 ADY7 Dublin, Ireland.; ‡ The Group of Applied Physics, Technological University Dublin, City Campus, Grangegorman Lower, D07 ADY7Dublin, Ireland; § School of Chemical and Biopharmaceutical Sciences, Technological University Dublin, City Campus, Grangegorman Lower, D07 ADY7 Dublin, Ireland

**Keywords:** fluorophores, BODIPY, luminescent downshifting, hybrid photovoltaic−thermal (PVT), solar energy conversion

## Abstract

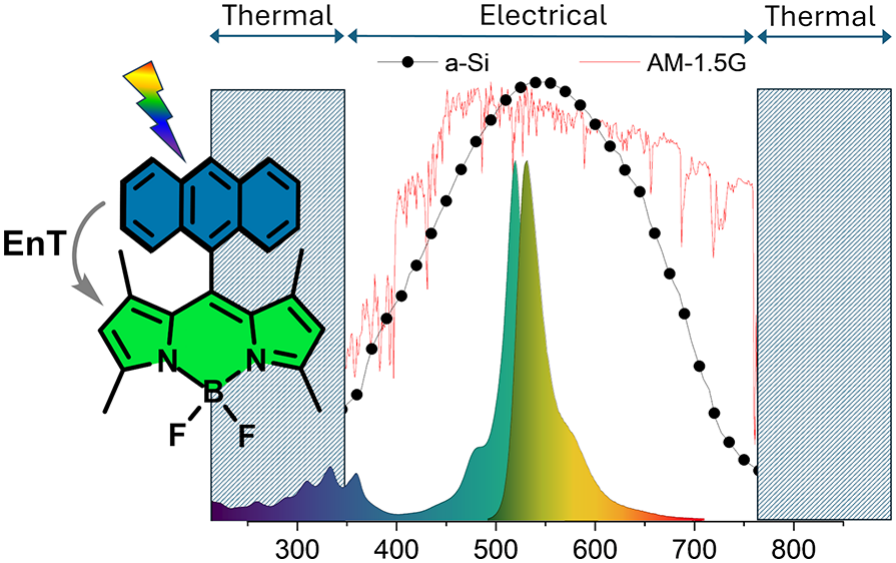

A library of boron dipyrromethene (BODIPY) compounds was studied to assess their efficacy as components of a working liquid in hybrid photovoltaic–thermal (PVT) systems. Two series of BODIPY dyes were investigated: series I included alkylBODIPYs with varying substitution patterns, while series II included 1,3,5,7-tetramethyl-substituted BODIPYs featuring electron-rich aromatic groups in the *meso* position, such as naphthalene, anthracene, and carbazole. Series II dyes were designed to exhibit luminescence downshifting due to enhanced UV absorption (300–400 nm) and excited-state energy transfer, leading to visible-region fluorescence under UV excitation. Samples of PVT liquids based on decalin and containing each individual BODIPY dye were tested on a standard a-Si solar cell to evaluate their impact on solar energy conversion efficiency. The thermal behavior of the working liquid and the cell during the illumination cycle was monitored, alongside the cell’s electrical characteristics. Energy conversion pathways and the overall effects of the dyes on the system performance were scrutinized. Results indicated that all BODIPY dyes enhanced both the electrical conversion efficiency (up to 2.41% increase) and thermal energy generation (up to 6.87%) compared to the solvent alone. These findings highlight the potential of BODIPY dyes to significantly improve the performance of PVT systems.

## Introduction

The traditional method of generating renewable energy from sunlight has been classically achieved through electrical energy generation through the implementation of a photovoltaic (PV) device or heat generation using photothermal (PT) panels. PV devices are capable of generating electricity through a process known as the PV effect, while PT systems are absorbing mainly infrared radiation.^1^ One challenge relating to the classical form of renewable electrical energy generation is that a large quantity of photons with unsuitable wavelengths for electrical generation result in thermal effects on the PV device or are lost through various mechanisms.^2^ This excess thermal energy leads to the PV cell heating up, which can hinder the efficiencies of the cell, with a rise of 1 °C leading to reductions of up to 0.50%.^3−7^ Reported methods for reducing these losses involve passive cooling through a fluid, such as water^8^ flowing behind a PV panel, as well as using radiative and antireflection cooling materials.^9,10^

A popular method of addressing the spectral mismatch between the incident solar radiation and the spectral response of the solar cell is to incorporate a luminescent species with known absorption properties at a particular (short) wavelength (e.g., in UV or in the blue region of the spectrum) and an emission spectrum at longer wavelengths, more suitable for the band-gap energy of the solar material in question. Spectral shifting methods such as this are known as luminescent downshifting (LDS).^11,12^ However, methods that absorb at higher wavelengths and emit at lower wavelengths are also possible. Such methods are referred to as upshifting methods.^13,14^

To increase the total quantity of solar energy conversion, hybrid photovoltaic–thermal (PVT) devices are being investigated.^2,15−19^ In a classical approach, in these devices the cooling aspect is provided by a heat-transfer fluid to reduce the temperature of the cell and thus improve the efficiencies. The fluid is flowing on the back of the PV cell, and no interaction between the working fluid and the incident solar radiation can take place. However, new approaches to this technology attempt to place the working fluid atop the solar cell, similar to previous attempts to incorporate a LDS solid layer.^15,17,20^ The added benefit of this configuration is that the working fluid can act as a spectral modulation fluid to alter the incident solar radiation to better adhere to the spectral response of the cell (much like classical LDS methods) while also acting as a heat-transfer fluid.^2^

Materials used for LDS in solar cells typically fall into one of the following categories: fluorescent dyes, quantum dots, and inorganic phosphors.^21^ Fluorescent dyes offer several advantages over other types of LDS materials, such as tunable optical properties, high fluorescence quantum yields, cost-effectiveness, and compatibility with various solar cell technologies. Additionally, fluorescent dyes are more environmentally friendly compared to materials containing heavy metals such as Cd, Pb, or Hg, which are common in quantum dots and inorganic phosphors.^22^ The synthesis and disposal of fluorescent dyes generally have lower environmental impacts. Due to their nontoxic nature, fluorescent dyes are easier to handle in compliance with regulatory standards and environmental requirements.^23^

Dyes that have been investigated as LDS materials for solar cells include coumarin,^24^ rhodamine,^25^ perylene,^26^ and perylenediimide dyes.^27^ These dyes were chosen based on their absorption and emission spectra, emission quantum yields, photostability, and compatibility with the materials used in solar cell devices. Exploring new functional dyes and optimizing their properties to further enhance the performance of solar cells through the LDS process is the subject of active research efforts.^28^

**Figure 1:**
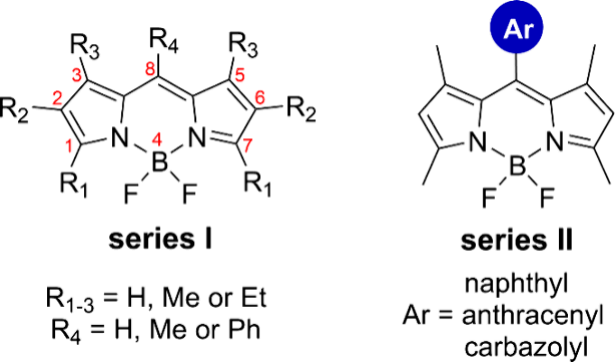
General structures of BODIPY dyes used in this work (series I and II) with IUPAC numbering.

Boron dipyrromethene (BODIPY) dyes (Figure 1) are popular fluorophores known for their exceptional optical properties, which include tuneable absorption and emission bands, high fluorescence quantum yields (Φ_Fl_), and remarkable extinction coefficients (up to 10^5^ M^–1^ cm^–1^).^29^ Their robust thermal stability and photostability further enhance their utility in diverse applications. A standout feature of BODIPY dyes is their structural versatility, allowing facile modification and the introduction of substituents and functional groups at various positions.^30^ This structural flexibility profoundly impacts the electronic properties of the chromophore, enabling precise modulation and fine-tuning of their optical characteristics to meet specific application requirements.

Due to these properties, BODIPY dyes have been extensively studied in fields such as imaging, sensing, and organic electronics.^31^ They have been utilized as light-harvesting components and electron acceptors in solar cell materials and devices, facilitating efficient charge separation and transport processes.^21^ However, to the best of our knowledge, the application of BODIPY dyes as components of LDS materials for solar cells and hybrid PVT devices has not yet been explored. Given that BODIPYs possess excellent optical properties aligning with the typical requirements for light-harvesting materials in LDS—namely, absorption in the UV (300–400 nm) and blue (400–500 nm) regions, high extinction coefficients, and fluorescence quantum yield values surpassing 0.9, along with requisite stability, relatively low cost, and scalability—it has prompted our investigation into their use in this application.

**Figure 2:**
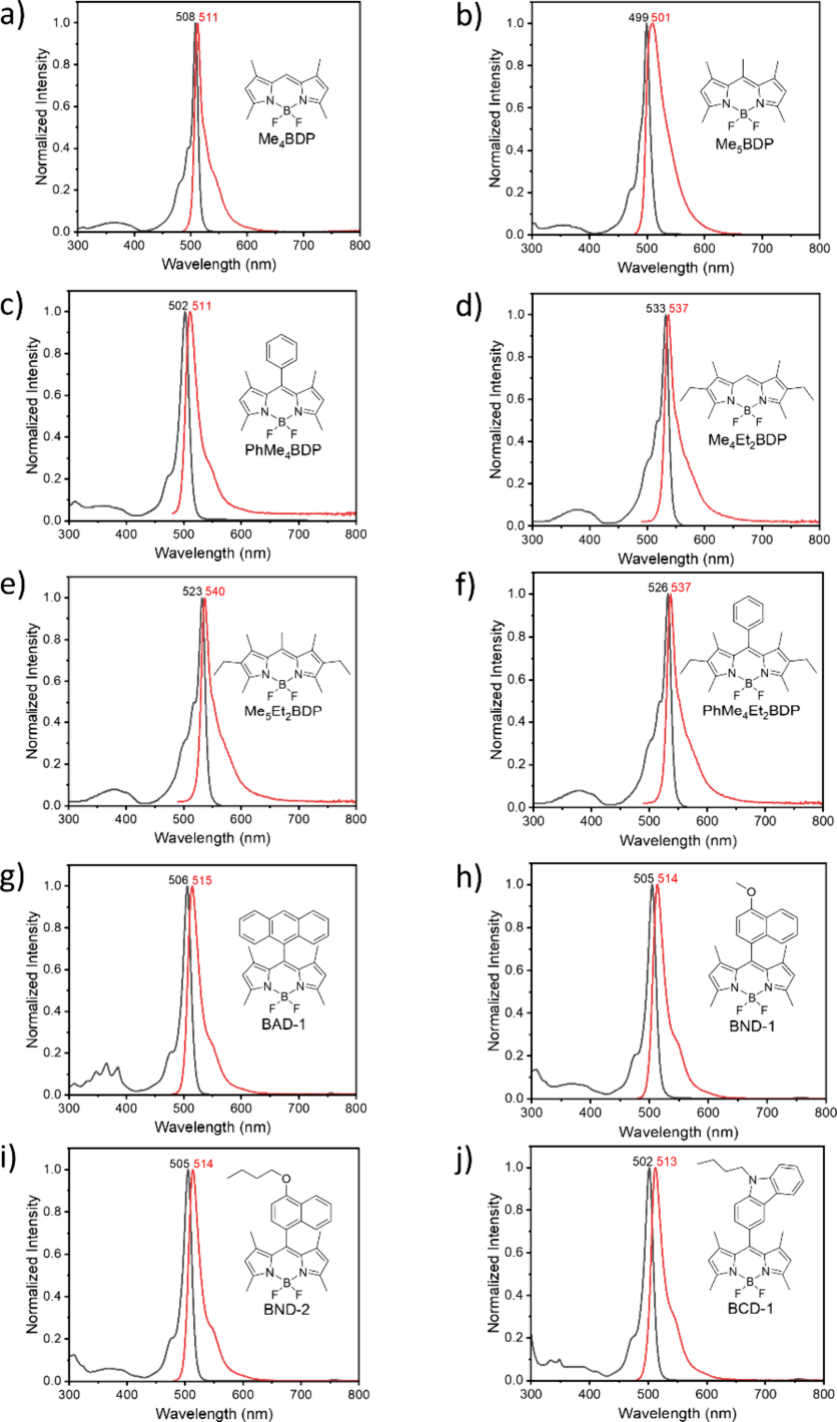
Absorption (a, c, e, g, and i) and emission (b, d, f, h, and j) spectra of the studied BODIPY dyes in a cyclohexane solution. The emission was excited at 470 nm.

In this work, we studied a library of 10 BODIPY compounds and evaluated their performances as components of a working liquid for a PVT system. Two general types of BODIPYs were involved: (1) alkyl-substituted BODIPY bearing methyl and/or ethyl groups in positions 1–8 (series I, Figure 1) and (2) 1,3,5,7-tetramethyl-substituted BODIPY bearing electron-rich aromatic groups in position 8, such as naphthalene, anthracene, and carbazole (series II, Figure 1). BODIPYs in series II were designed to possess enhanced absorption in the UV region (300–400 nm) due to the presence of aromatic units. These dyes undergo energy transfer in the excited state, emitting fluorescence in the visible region upon excitation in the UV region. To ensure high emission quantum yields, the studied compounds were dissolved in a solvent with low polarity, which prevents photoinduced electron transfer (PET) between the aromatic group and the BODIPY chromophore, thereby preventing fluorescence quenching. Solubility in nonpolar solvents was optimized by introducing additional alkyl groups (*n*-butyl groups) to the *meso*-aryl subunit. A working PVT liquid based on decalin as a solvent containing these individual BODIPY dyes was tested using a standard a-Si solar cell to evaluate their effects on the solar energy conversion efficiency. The obtained results demonstrate enhanced solar energy conversion through increased electrical energy and thermal energy generation, highlighting the potential of these fluorescent compounds for the next generation of PVT systems.

## Experimental Section

### General Procedures

^1^H and ^13^C NMR spectra were recorded on a Bruker Avance III 500 MHz spectrometer. Chemical shifts are reported as parts per million relative to tetramethylsilane and referenced to residual solvent peaks of CDCl_3_ (δ 7.26 ppm). Multiplicity assignments are abbreviated as follows: s = singlet, d = doublet, t = triplet, q = quartet, dt = doublet of triplets, dq = doublet of quartets, ddd = doublet of doublet of doublets, and m = multiplet.

UV–vis absorption spectra were recorded in solutions using Shimadzu UV-1900i and PerkinElmer Lambda 900 UV/vis/near-IR spectrometers (1-cm-path-length quartz cell). Fluorescence emission spectra were measured by using FluoroMax-4 spectrometer. Emission quantum yields of the compounds were measured relative to the fluorescence of fluorescein disodium salt as a standard (Φ_em_ = 0.95 in 0.1 M NaOH).^32^ Sample concentrations were chosen to obtain an absorbance of 0.03–0.07 at the excitation wavelength; at least three measurements were performed for each sample. Accurate mass measurements (HRMS) were performed using an Agilent 6546 QToF (quadrupole time-of-flight) mass spectrometer with an Agilent 1260 Infinity Prime II quaternary pump; chromatography was effected via a 50 mm Agilent Poroshell 2.7 μm C18 column. Liquid chromatography mobile phases and buffers were as follows: A, H_2_O with 0.1% formic acid. B, acetonitrile with 0.1% formic acid. Gradients were 90% A/10% B (time = 0 min), 10% A/90% B (time = 5 min), and 10% A/90% B (time = 10 min). Data analysis was carried out using Agilent *Masshunter* software.

### Synthesis and Characterization

The handling of all air/water-sensitive materials was carried out using standard high-vacuum techniques. Dichloromethane (DCM) was distilled from CaH_2_. All other solvents were used as commercially supplied. Analytical thin-layer chromatography (TLC) was performed using silica gel 60 (fluorescence indicator F254, precoated sheets, 0.2 mm thick, 20 cm × 20 cm; Merck) plates and visualized by UV irradiation (λ = 254 nm). Column chromatography was carried out using Fluka silica gel 60 (230–400 mesh).

Trifluoracetic acid (TFA), phosphoryl chloride, 2,3-dichloro-5,6-dicyano-1,4-benzoquinone (DDQ), boron trifluoride diethyl etherate, *N*,*N*-diisopropylethylamine (DIPEA), 2,4-dimethyl-1*H*-pyrrole, 3-ethyl-2,4-dimethyl-1*H*-pyrrole, and 4-methoxy-1-napthaldehyde were purchased from Merck. 4-Butoxy-1-napthaldehyde^33^ and 9-butyl-9*H*-carbazole-3-carbaldehyde^34^ were prepared as described previously. Compounds Me_4_BDP and Me_4_Et_2_BDP were prepared according to the method reported by Wu and Burgess.^35^ Compounds Me_5_BDP and Me_5_Et_2_BDP were prepared according to the method reported by Bard and coauthors.^36^

Compounds PhMe_4_BDP, PhMe_4_Et_2_BDP, BAD-1, BND-1, BND-2, and BCD-1 were synthesized by applying the following procedure. Corresponding pyrrole (2,4-dimethylpyrrole or 3-ethyl-2,4-dimethylpyrrole, 10.72 mmol, 1 equiv) and aldehyde (5.37 mmol, 0.5 equiv) were dissolved in dry DCM (60 mL) and deoxygenated by bubbling N_2_ gas through the solution for 10 min. TFA (20 μL, 0.5 mmol) was then added to the reaction and stirred under N_2_ for 1 h. The reaction progress was monitored by TLC analysis using hexane–DCM (1:1 by volume) as an eluent. DDQ (1.22 g, 5.37 mmol) was dissolved in DCM (∼10 mL), added to the reaction mixture, and stirred under N_2_ for 1 h. The reaction was then quenched by adding 100 mL of H_2_O. The resulting mixture was transferred to a separating funnel and extracted with DCM (3 × 50 mL). Combined extracts were dried over Na_2_SO_4_ and evaporated under a vacuum.

The obtained solid residue was redissolved in dry DCM (50 mL). To this solution was added DIPEA (1.73 g, 13.4 mmol, 2.5 equiv), and then BF_3_·Et_2_O (2.29 g, 16.1 mmol, 3 equiv) was added under N_2_ and stirred at room temperature for 2 h. The reaction progress was monitored by TLC analysis. The reaction was then quenched with H_2_O (100 mL), and the resulting mixture was extracted with DCM (3 × 50 mL). Combined extracts were dried over Na_2_SO_4_ and evaporated under a vacuum. The crude product was purified by column chromatography on a hexane-packed silica column using hexane–DCM (1:1 by volume) as an eluent.

Compound PhMe_4_BDP: orange solid, 29% yield. Analytical data are in agreement with the literature.^37^

Compound PhMe_4_Et_2_BDP: orange solid, 18% yield. Analytical data are in agreement with the literature.^37^

Compound BAD-1: orange solid, 40% yield. Analytical data are in agreement with the literature.^38^

Compound BND-1: dark-green crystalline solid, 25 % yield. ^1^H NMR (400 MHz, CDCl_3_): δ 8.24 (d, 1H), 7.64 (d, 1H), 7.47–7.32 (m, *J* = 24.3, 8.2, 6.8, and 1.3 Hz, 2H), 7.21 (s, 1H), 6.84 (d, *J* = 7.9 Hz, 1H), 5.86 (s, 2H), 4.00 (s, 3H), 2.51 (s, 6H), 1.05 (s, 6H). ^13^C NMR (101 MHz, CDCl_3_): δ 155.23, 154.32, 142.06, 139.54, 131.59, 131.42, 126.65, 125.05, 124.83, 124.60, 123.68, 123.41, 121.11, 119.98, 102.63, 76.19, 54.59, 13.61, 13.01. HRMS (ESI). Found: *m*/*z* 405.1950. Calcd for C_24_H_25_BF_2_N_2_O [(M + H)^+^]: *m*/*z* 405.1950.

Compound BND-2: red crystalline solid, 7% yield. ^1^H NMR (400 MHz, CDCl_3_): δ 8.33 (d, *J* = 9.6 Hz, 1H), 7.71 (d, *J* = 7.9 Hz, 1H), 7.54–7.38 (m, *J* = 24.5, 8.2, 6.8, and 1.3 Hz, 2H), 6.90 (d, *J* = 7.2 Hz, 1H), 5.93 (s, 2H), 4.20 (t, *J* = 6.4 Hz, 2H), 2.58 (s, 6H), 2.25 (s, 1H), 2.01–1.92 (m, *J* = 12.6 and 6.5 Hz, 2H), 1.71–1.59 (m, 2H), 1.13 (s, 6H), 1.06 (t, *J* = 7.4 Hz, 3H). ^13^C NMR (101 MHz, CDCl_3_): δ 155.74, 155.30, 143.11, 140.69, 132.62, 132.46, 127.60, 126.13, 125.72, 124.67, 124.11, 122.23, 120.97, 104.35, 77.22, 68.03, 31.36, 19.52, 14.63, 14.07, 14.00, 11.27. HRMS (ESI). Found: *m*/*z* 447.2418. Calcd for C_27_H_30_BF_2_N_2_O [(M + H)^+^]: *m*/*z* 447.2419.

Compound BCD-1: dark-red crystalline solid, 7% yield. ^1^H NMR (400 MHz, CDCl_3_): δ 8.07 (d, *J* = 7.8 Hz, 1H), 7.99 (d, *J* = 1.5 Hz, 1H), 7.56–7.45 (m, 3H), 7.35–7.26 (m, 2H, overlap with solvent), 5.98 (s, 2H), 4.36 (t, *J* = 7.3 Hz, 2H), 2.58 (s, 6H), 1.97–1.85 (m, 2H), 1.57–1.39 (m, 2H), 1.31 (s, 6H), 0.99 (t, *J* = 7.4 Hz, 3H). ^13^C NMR (101 MHz, CDCl_3_): δ 155.20, 143.52, 143.35, 140.92, 140.70, 132.43, 126.39, 125.41, 125.22, 123.40, 122.70, 121.17, 120.67, 120.12, 119.41, 109.46, 109.20, 43.28, 31.22, 20.76, 14.76, 14.03. HRMS (ESI). Found: *m*/*z* 470.2577. Calcd for C_29_H_31_BF_2_N_3_ [(M + H)^+^]: *m*/*z* 470.2579.

### PVT Experiments

The experimental setup used to investigate the PVT system is presented in Figure S8.

An a-Si solar cell (SOLEMS 05/020/015E0A) was positioned at ∼1.5 mm behind the quartz cuvette, and its electrical properties were evaluated at 5 min intervals throughout the entire illumination process. The electrical characteristics were recorded using an Ossila Xtralien X200 sourcemeter, which provides a voltage measurement with an uncertainty of ±10 mV and a current measurement with an uncertainty of ±10 nA. The unobstructed (i.e., the bare) a-Si solar cell was evaluated under the illumination cycle to determine the baseline characteristics of the solar cell under AM1.5G conditions. The illumination cycle consists of 100 min of direct illumination, followed by a cooling period (usually 60 min) to allow the PV and the fluid to return to room temperature.

The inclusion of various working fluids was performed by transferring ∼3 mL to the cuvette. With the fluid added, the sample was deoxygenated by bubbling argon through the solution for 5 min and then sealed with Parafilm. A Pico USB TC08 Temperature Data Logger was used to collect the signal from 3 K-type thermocouples configured to record the temperature of the cell (*T*_cell_) and working fluid (*T*_fluid_) and the room temperature throughout the entire experimental process at 1 s intervals.

### PVT Energy Conversion Calculation

The reverse saturation current density *J*_00_ of the modeled a-Si PV cell was determined using eq 1:
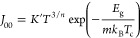
1where *K*′, *n*, and *m* are empirical constants, *E*_g_ represents the band-gap energy, *k*_B_ is the Boltzmann constant, and *T*_c_ is the solar cell temperature; the numerical values of these constants can be found in previous works.^1,15^

The short-circuit current density *J*_SC_ was determined using the following equation:
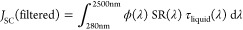
2where SR(λ) represents the spectral response of the investigated amorphous silicon cell and ϕ(λ) represents the transmitted irradiance that is incident upon the PV cell after passing through the working fluid. This transmitted irradiance is represented in the model using the “transmitted” photons, which are composed of both transmitted and LDS photons that impinge on the PV cell. *V*_OC_ represents the open-circuit voltage, and it is determined via
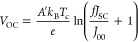
3where *e* is the fundamental unit of charge, *f* is the concentration factor, and *A*′ is the ideality factor. The fill factor (FF) is determined by
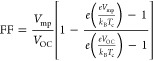
4where *V*_mp_ is the maximum voltage derived at the maximum power point of the *I*–*V* response curve, which can be approximated by
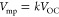
5where *k* is typically between 0.7 and 0.8. The electrical power output derived by the a-Si PV cell with an accompanying working fluid can be determined by multiplying the corresponding *V*_OC_ and FF for *J*_SC_(filtered) as follows:
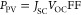
6The thermal power output of the PVT system when equipped with working fluid (*P*_th_) is attained by determining the outgoing spectrum of light that is directed toward the solar cell after spectral modulation has occurred within the simulation. This value is calculated as
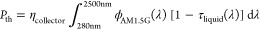
7where the absorption of the working fluids is quantified by the 1 – τ_liquid_(λ) term. In this scenario, we assume that absorption is determined by the outward spectrum. Assumptions are also made relating to the collector efficiency, η_collector_, of the thermal component within the PVT system, which is assumed to be 67%.^17^ The dynamic competition between the percentage of the solar irradiance partitioned into usable thermal and electrical energy for each working fluid was revealed through the following expression:
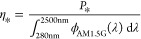
8where P_*_ represents the thermal (*P*_th_) or electrical (*P*_PV_) power output of the PVT system and η_*_ is the energy conversion percentage for this type of energy.

To evaluate whether a working fluid can convert solar energy into electrical and thermal energy more efficiently within a PVT system, a merit function is implemented.
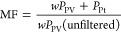
9Here *P*_PV_ and *P*_PV_(unfiltered) are the filtered and unfiltered (i.e., no cuvette) electrical power outputs of the silicon cell, and *w* is the worth factor of electricity to thermal energy.

Classically, heat-transfer fluids, such as the ones employed in this study, are evaluated using a 3:1 worth factor of electricity to thermal energy. All values used for eqs 1–9 can be seen in previous works.^1,15^

## Results and Discussion

### Molecular Design and Synthesis

To investigate and optimize the performance of BODIPY dyes as components of PVT liquids, a range of structures with different substitution patterns was selected. The chemical structures and optical properties of the investigated compounds are listed in Figure 2.

Alkyl substitution at positions 1–7 of the BODIPY chromophore (Figure 1) significantly influences its electronic and photophysical properties.^39^ The energies of the BODIPY HOMO orbitals increase gradually when going from unsubstituted BODIPY to hexaalkyl-substituted ones, while the HOMO–LUMO gap is steadily decreasing.^40^ Thus, increasing the number of alkyl substituents in pyrrolic positions (1–3 and 5–7) of the BODIPY core typically induces a bathochromic shift. The introduction of alkyl groups can also enhance the fluorescence quantum yield and solubility of BODIPY derivatives, making them more efficient in fluorescence-based applications.^41^ Additionally, variation in the alkyl substitution pattern enables tuning of the redox potential of BODIPY derivatives, impacting their behavior in electron-transfer processes.^42^ Substitution at the *meso* position (position 8, Figure 1) of the BODIPY core also affects its electronic properties. *Meso*-substituted BODIPYs exhibit altered absorption and emission spectra, influenced by changes in the electronic distribution within the chromophore. This substitution can lead to a bathochromic shift in absorption, modifying the molar extinction coefficient and fluorescence quantum yield by restricting intermolecular rotation.^29^

For this study, BODIPY derivatives containing either only methyl substituents at positions 1, 3, 5, and 7 (compounds Me_4_BDP, Me_5_BDP, and PhMe_4_BDP) or additional ethyl groups at positions 2 and 6 (compounds Et_2_Me_4_BDP, Et_2_Me_5_BDP, and PhEt_2_Me_4_BDP) were selected. This choice was based on the availability of the corresponding pyrrole precursors (2,4-dimethylpyrrole and 3-ethyl-2,4-dimethylpyrrole, respectively) and optimized synthetic protocols for the preparation of the target compounds.

Electron-rich aromatic groups such as naphthalene, anthracene, and carbazole were introduced in series II with the purpose of enhancing absorption in the UV region (300–400 nm) for exploiting the potential LDS performance of these dyes. Butyl chains were introduced in compounds BND-2 and BCD-1 to further improve the solubilities of these dyes in nonpolar solvents.

The synthesis of Me_4_BDP, Me_5_BDP, Et_2_Me_4_BDP, and Et_2_Me_4_BPD dyes was performed by following a previously published method relying on corresponding alkyl-substituted pyrrole aldehydes as starting materials.^43^ Compounds PhMe_4_BDP, PhEt_2_Me_4_BDP, BAD-1, BND-1, BND-2, and BCD-1 were synthesized using an acid-catalyzed condensation of alkylpyrroles with aromatic aldehydes.^29^ Purity of all compounds was verified by NMR and HRMS, and the details can be found in Figures S1–S4. The obtained compounds are well-soluble in common organic solvents such as DCM, toluene, acetonitrile, and cyclohexane. They exhibited high photostability and thermal stability both in solution and in the solid state, indicating their potential utility in PVT applications requiring robust performance.

### Optical Absorption and Fluorescence Properties

The absorption and fluorescence spectra of the studied BODIPY compounds are illustrated in Figure 2, and the molar extinction coefficients and fluorescence quantum yield values measured in cyclohexane are summarized in Table 1.

**Table 1:** Spectroscopic Data for the Studied Dyes in Cyclohexane (ε_r_ = 2.02)

	λ_max_ (nm)^a^			
compound	absorption	emission	ε × 10^4^(M^–1^ cm^–1^)	Φ_Fl_^b^
Me_4_BDP	508	511	9.23	0.9955
Me_5_BDP	499	510	7.43	0.8726
PhMe_4_BDP	502	511	8.01	0.4213
Et_2_Me_4_BDP	533	537	7.70	0.9614
Et_2_Me_5_BDP	523	540	8.65	0.9256
PhEt_2_Me_4_BDP	526	537	8.72	0.5893
BAD-1	506	515	8.51	0.7623
BND-1	505	514	8.43	0.99
BND-2	505	514	8.72	0.9806
BCD-1	502	513	7.47	0.6512

a Concentration of the samples: 5 × 10^–6^ M.

b The fluorescence quantum yields were measured using fluorescein disodium salt as a standard (Φ_Fl_ = 0.95 in 0.1 M NaOH).

The simplest studied compound, 1,3,5,7-tetramethylBODIPY (Me_4_BDP), absorbs at 506 nm, displaying a sharp absorption peak (Figure 2a). It exhibits a small Stokes shift and, upon excitation, emits fluorescence at a maximum of 511 nm and a fluorescence quantum yield of 0.99. A derivative bearing an additional methyl group in the *meso* position, Me_5_BDP, exhibits absorption and emission maxima slightly blue-shifted compared to Me_4_BDP. Its extinction coefficient and Φ_Fl_ values are also reduced (Table 1). The emission spectrum of Me_5_BDP is characterized by a broader spectral width, reflecting structural variation with an additional methyl group. A *meso*-phenyl-substituted analogue, PhMe_4_BDP, features a red-shifted absorption peak compared to Me_5_BDP, at 502 nm, attributable to the conjugation of the phenyl group in the BODIPY aromatic system. Its emission, however, shows a reduced quantum yield of 0.42, which was attributed to increased nonradiative decay associated with intermolecular rotation of the phenyl group.^29^

Introducing ethyl groups at positions 2 and 6 of the BODIPY core, such as in Et_2_Me_4_BDP, results in a red shift in both absorption and emission by almost 30 nm compared to Me_4_BDP due to the increased HOMO–LUMO gap.^40^ Despite this shift, the fluorescence quantum yield remains high (0.96), indicating that the introduction of ethyl groups at these positions does not significantly affect the radiative decay rates. Introducing an extra methyl group in the *meso* position (Et_2_Me_5_BDP) leads to a blue shift in the absorption by 10 nm, while the emission maximum and Φ_Fl_ values are almost unaffected. Alternatively, introduction of the phenyl group (PhEt_2_Me_4_BDP) leads to a drop in Φ_Fl,_ similar to that observed for the case of PhMe_4_BDP.

The introduction of larger aromatic fragments to the *meso* position, in BAD, BND, and BCD dyes, leads to a substantial increase in the Φ_Fl_ values, reaching 0.99 in the case of BND-1 due to the hindered intermolecular rotation. The observed fluorescence emission is concentration-dependent: a hypochromic shift of up to 20 nm was observed for BAD-1 and other studied dyes when the dye concentration was increased from 10^–5^ to 10^–3^ (Figure S5); a similar behavior was previously observed for BODIPYs forming *J* aggregates.^44^ It should be noted that the formation of these aggregates does not cause concentration quenching, as was confirmed by measurement of the absolute fluorescence quantum yield. When the concentration was increased from 10^–5^ to 10^–3^, the Φ_Fl_ value was only slightly reduced from 0.759 to 0.725 (Table S2).

Selective excitation of the anthracene subunit in BAD-1 at 350 nm leads to emission exclusively from the BODIPY subunit (Figure S6a). A similar behavior was observed for BND-1–2 and BCD-1 dyads upon selective excitation of the naphthalene and carbazolyl subunits, respectively (Figure S5, panels b–e). As was shown in a previous work, excitation of the aromatic subunit in the UV region forms singlet excited states localized on this subunit, which is followed by an ultrafast energy transfer (rate constant > 10^12^) to the BODIPY subunit.^40^

BODIPYs containing electron-donating aromatic substituents such as anthracenyl^45,46^ and pyrenyl^47,48^ groups exhibit PET from the aromatic group to the BODIPY subunit, forming charge-transfer (CT) states, which render such dyes non-fluorescent. The electron transfer process is typically pronounced in polar solvents (e.g., alcohols), which stabilizes the CT states through dipole–dipole interactions.^49^ When the solvent polarity is changed, the shape and maxima of the absorption and emission bands of BODIPYs belonging to series I undergo little change, as is shown in Figure S7 in an example of PhMe_4_BPD. However, in the case of series II, the emission properties are significantly affected by the solvent polarity. As shown in Figure S7d for BAD-1, in polar acetonitrile, a new broad and structureless band appears at 620 nm, accompanied by a remarkable drop in the emission intensity and quantum yield to 0.006 (Table S2). The emission spectrum observed for BAD-1 in acetonitrile can be interpreted as a mixture of fluorescence from the LE and CT states, formed via intramolecular electron transfer from the anthracene subunit to the BODIPY subunit. Conversely, in a less polar solvent, such as toluene, this dye shows strong fluorescence (Φ_Fl_ = 0.92) because, under these conditions, the energy of the CT state is higher than that of S_1_,^50^ rendering the charge transfer process inefficient.

To block the PET process and maximize the fluorescence quantum yield, these dyes should be used in a nonpolar solvent, such as cyclohexane. However, its boiling point (81 °C) does not allow for application in the PVT liquid. Decalin (a mixture of *cis* and *trans* isomers) was chosen in this study due to its structural similarity to cyclohexane and low polarity (dielectric constant ε_r_ = 2.23). At the same time, its ability to dissolve a wide range of substances, chemical inertness, and high boiling point (186 °C) makes it an effective heat-transfer fluid in processes requiring stable and high-temperature operation.

### Thermal Behavior of BODIPY-Based Fluids

**Figure 3:**
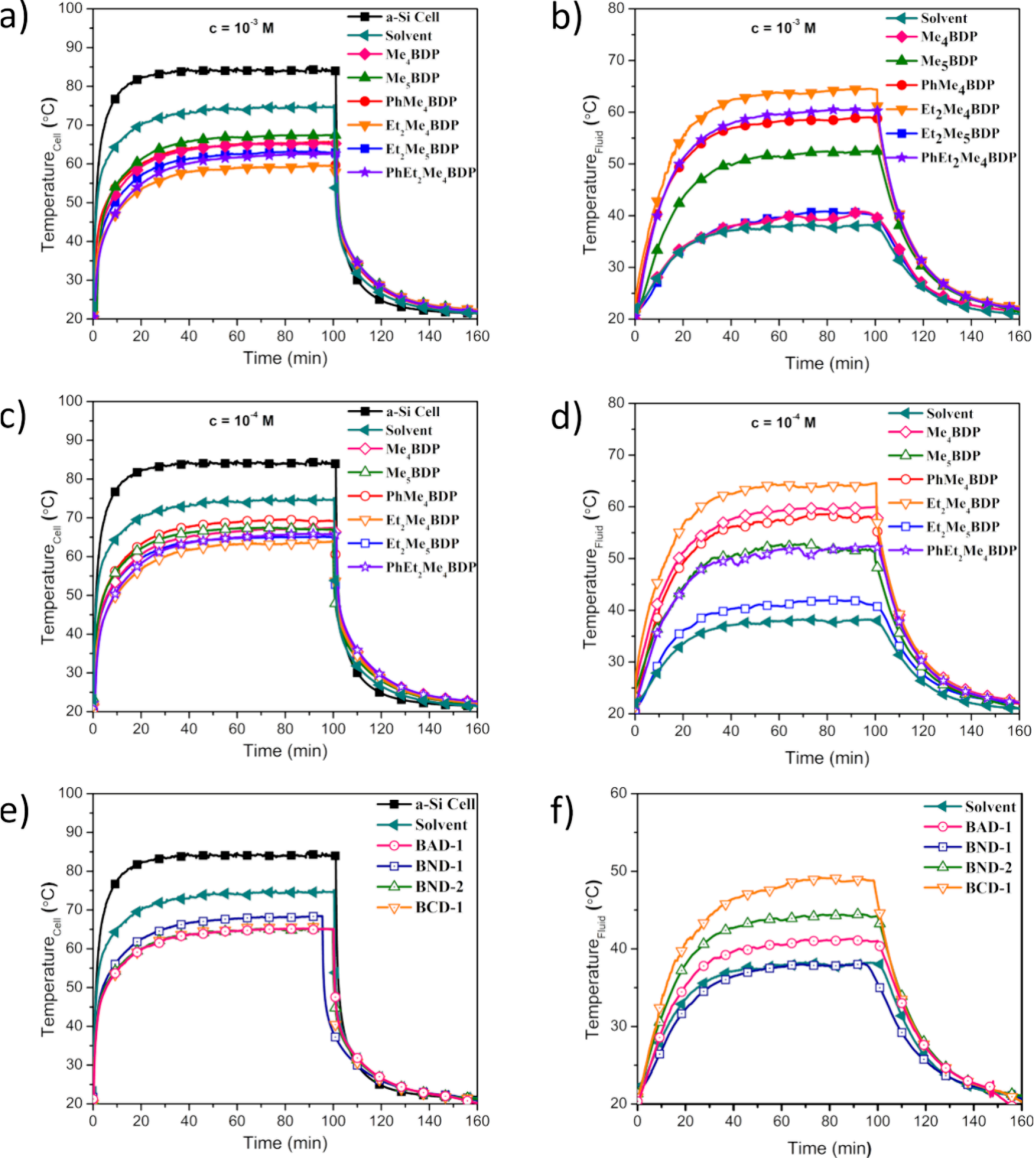
Temperatures recorded for both *T*_Cell_ (a, c, and e) and *T*_Fluid_ (b, d, and f) for all proposed PVT systems. Temperatures were allowed to plateau to ensure that the maximum temperatures of *T*_Cell_ and *T*_Fluid_ were reached.

During the illumination cycle, the temperatures of both the working fluid, i.e., a solution of BODIPY in decalin, and the a-Si cell (*T*_Fluid_ and *T*_Cell_, respectively) were recorded, as presented in Figure 3. The bare a-Si cell (i.e., the unobstructed PV cell) reached a peak temperature of ∼85 °C (black squares, Figure 3a,c,e). However, the introduction of a solvent base fluid into the PVT system reduced the maximum *T*_Cell_ to ∼75 °C. This indicates that the base fluid alone effectively lowers the PV cell temperature placed behind it. Thereby, this can mitigate the adverse effects associated with the reduction of the PV efficiency when it operates at high temperatures. Simultaneously, *T*_Fluid_ was monitored every second during the illumination cycle (Figure 3b,d,f). Thus, pure solvent exhibited a maximum *T*_Fluid_ of ∼38 °C.

The influence of the various BODIPY dyes on the maximum temperature achieved by the cell or by the fluid is summarized in Table 2. These temperatures are important because, as the temperature variation reaches a plateau (Figure 3), they represent the temperatures at which the PVT systems will operate. From Figure 3 and Table 2, it can be seen that the chemical composition and concentration (see parts a and c of Figure 3 for 10^–3^ and 10^–4^ M, respectively) are playing an important role in the thermal behavior of the PVT system. For the 10^–3^ M concentrations, the best-performing dye in terms of reduction in *T*_cell_ was the Et_2_Me_4_BDP (orange inverted triangle, Figure 3a) dye, which determined a reduction of 25.38 °C compared to the temperature of a bare a-Si cell. This result represents the largest reduction induced in *T*_cell_ by a dye compared to those tested here. With a reduction of 17.48 °C on *T*_cell_, the Me_5_BDP dye had the lowest impact on reducing the PV operation temperature. Independent of their concentrations, all BODIPY dyes led to a reduction in *T*_cell_ larger than the solvent alone. This suggests that, by leading to a lower operation temperature of the PV cell, the BODIPY cells can extend the PV lifetime and long-term performance.

**Table 2:** Maximum Temperatures Measured for Both the a-Si Cell and the Working Fluid throughout the Illumination Cycle

series	compound	concentration (M)	*T*_cell_max_ (°C)	*T*_fluid_max_ (°C)	Δ*T*_cell_max_^a^ (°C)	Δ*T*_fluid_max_^b^ (°C)
series I	bare a-Si cell	10^–3^	84.95			
	decalin		75.14	38.29	–9.81	
	Me_4_BDP		65.34	40.76	–19.61	2.47
	Me_5_BDP		67.47	52.51	–17.48	14.22
	PhMe_4_BDP		65.73	59.03	–19.22	20.74
	Et_2_Me_4_BDP		59.57	64.55	–25.38	26.26
	Et_2_Me_5_BDP		63.28	40.82	–21.67	2.53
	PhEt_2_Me_4_BDP		62.77	60.54	–22.18	22.25
	Me_4_BDP	10^–4^	67.48	59.99	–17.47	21.70
	Me_5_BDP		67.51	52.88	–17.44	14.59
	PhMe_4_BDP		69.63	58.59	–15.32	20.30
	Et_2_Me_4_BDP		63.96	64.61	–20.99	26.32
	Et_2_Me_5_BDP		65.18	41.96	–19.77	3.67
	PhEt_2_Me_4_BDP		65.88	52.48	–19.07	14.19
series II	BAD-1	10^–4^	65.24	41.30	–19.71	3.01
	BND-1		68.49	38.09	–16.46	–0.20
	BND-2		65.26	44.47	–19.69	6.18
	BCD-1		65.79	49.20	–19.16	10.91

a Δ*T*_cell_max_ compares the maximum temperature difference between the measured compound and the bare a-Si cell.

b Δ*T*_fluid_max_ compares the maximum temperature difference between the measured compound and the pure solvent. The uncertainty in temperature measurements was ±0.01 °C.

Positive results were gathered for the *T*_Fluid_ measurements for these dyes, with all dyes successfully achieving a higher maximum *T*_Fluid_ during the illumination cycle compared to the pure solvent (Figure 3b,d,f and Table 2). Me_4_BDP and Et_2_Me_5_BDP exhibited minor increases in *T*_Fluid_ with maximum *T*_Fluid_ values of 2.47 and 2.53 °C larger than the temperature reached by the pure solvent (decalin). The best-performing dye of this concentration with respect to *T*_Fluid_ was EtMe_4_BDP, which exhibited a temperature difference of 26.26 °C.

Similar positive results were obtained from the 10^–4^ M concentration of series I dyes, but not to the same extent as the 10^–3^ M concentration.

Similar temperature reductions were observed with the incorporation of series II dyes of BAD-1, BCD-1, BND-1, and BND-2 at the 10^–4^ M concentration, as illustrated in Figure 3e and Table 2, but their performances do not overcome the best-performing dyes from series I.

### Influence of BODIPY Dyes on the Electrical Characteristics of a-Si Cell in a PVT System

**Figure 4:**
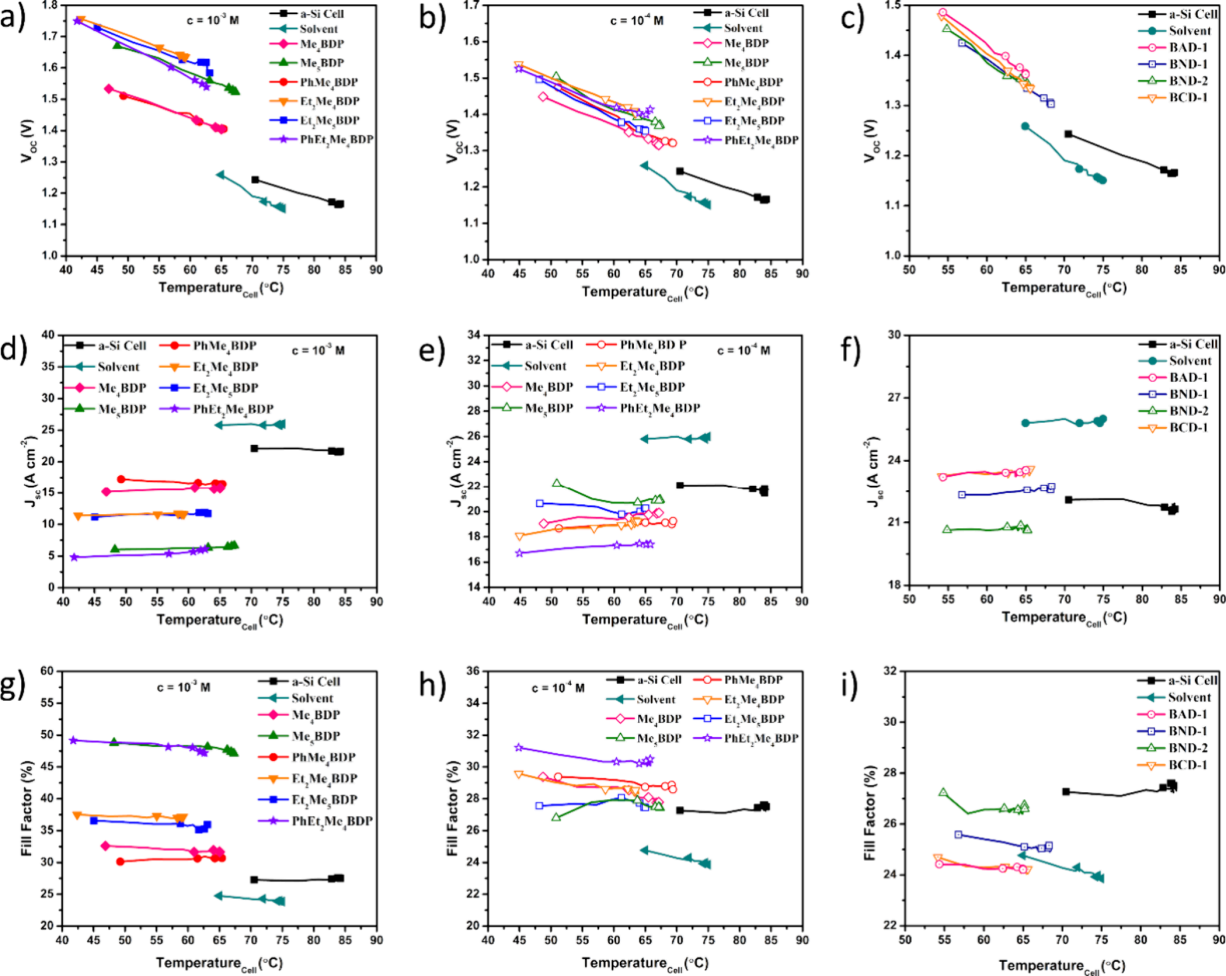
Various electrical characteristics recorded from the illuminated solar cell at 5-min intervals. *V*_OC_ (a–c), *J*_SC_ (d–f), and FF (g–i) are presented for the various dyes and concentrations investigated.

An immediate observation (which can be made from Figure 4a–c and Table 3) is that the inclusion of dyes investigated for use in PVT systems leads, initially (i.e., when the temperature of the cell is still at room temperature), to a rise in the *V*_OC_ value compared with the bare a-Si cell. However, the expected trend of a reduction in *V*_OC_ due to a rise in temperature is also observed for all dyes as well as for the pure solvent.

**Table 3:** Electrical Characteristics Measured for the a-Si Solar Cell When Coupled with the Various Compounds in a Hybrid PVT Configuration at the Initial Temperature^a^

series	compound	concentration (M)	*V*_OC,max_ (V)	*J*_SC_ (A cm^–2^)	FF_max_ (%)	*P*_max_ (μW)	*T*_cell_inital_ (°C)
	bare a-Si cell		1.24	21.73	27.68	74.89	21.14
	decalin		1.26	25.87	24.77	79.95	21.08
series I	Me_4_BDP	10^–3^	1.53	15.71	32.59	75.86	20.65
	Me_5_BDP		1.67	6.47	48.80	49.12	21.46
	PhMe_4_BDP		1.51	16.63	30.94	78.47	21.60
	Et_2_Me_4_BDP		1.76	11.68	37.52	75.16	21.02
	Et_2_Me_5_BDP		1.73	11.73	36.58	70.69	22.19
	PhEt_2_Me_4_BDP		1.75	5.71	47.74	45.16	20.48
	Me_4_BDP	10^–4^	1.45	19.73	29.38	81.06	21.86
	Me_5_BDP		1.50	20.97	27.92	89.67	22.44
	PhMe_4_BDP		1.47	19.07	29.38	80.87	20.54
	Et_2_Me_4_BDP		1.54	18.99	29.58	82.30	21.52
	Et_2_Me_5_BDP		1.50	20.15	28.08	85.11	21.50
	PhEt_2_Me_4_BDP		1.53	17.40	31.22	79.55	22.16
series II	BAD-1	10^–4^	1.49	23.45	24.42	84.98	21.25
	BND-1		1.42	22.57	25.58	80.08	21.71
	BND-2		1.45	20.71	27.22	79.38	20.77
	BCD-1		1.48	23.48	24.69	83.50	20.16

a The electrical characteristics were recorded using an Ossila Xtralien X200 sourcemeter, which provides a voltage measurement uncertainty of ±10 mV and a current uncertainty of ±10 nA.

The bare a-Si cell was found to have a maximum *V*_OC_ value at 70.50 °C of 1.24 V, while the introduction of the solvent produced a maximum *V*_OC_ value of 1.26 V. The majority of 10^–3^ M concentration dyes (Figure 4a) had a higher initial *V*_OC_ value than the bare a-Si cell, but they experienced the similar rates of change (Table 4) at an average of −7.79 ± 1.13 mV °C^–1^. Of these dyes, the Et_2_Me_4_BDP dye had a maximum *V*_OC_ value of 1.77 V, the highest *V*_OC_ value observed for any dye or concentration.

**Table 4:** Electrical Characteristics Measured for the a-Si Solar Cell When Coupled with the Various Compounds in a Hybrid PVT Configuration at Its Final Maximum Temperature^a^

series	compound	concentration (M)	d*V*_OC_/d*T* (mV °C^–1^)	dFF/d*T* (% °C^–1^)	d*P*/d*T* (μW °C^–1^)	*T*_cell_final_ (°C)	Δ*T*_cell_ (°C)
	bare a-Si Cell		–5.70	2.26	–4.08	83.86	62.72
	decalin		–10.7	–8.42	–8.72	74.65	53.57
series I	Me_4_BDP	10^–3^	–7.21	–4.55	–3.17	65.31	44.66
	Me_5_BDP		–7.89	–8.68	–6.33	67.45	45.99
	PhMe_4_BDP		–6.79	3.06	–4.53	65.59	43.99
	Et_2_Me_4_BDP		–7.37	–2.61	–2.59	59.43	38.41
	Et_2_Me_5_BDP		–7.30	–7.07	–2.43	62.99	40.80
	PhEt_2_Me_4_BDP		–10.2	–10.13	1.63	62.68	42.20
	Me_4_BDP	10^–4^	–7.20	–8.38	–4.67	67.44	45.58
	Me_5_BDP		–7.42	0.83	–5.87	66.95	44.51
	PhMe_4_BDP		–8.27	–3.99	–4.65	69.12	48.58
	Et_2_Me_4_BDP		–7.10	–5.22	–2.91	63.91	42.39
	Et_2_Me_5_BDP		–8.07	–0.67	–5.72	65.11	43.61
	PhEt_2_Me_4_BDP		–5.23	–4.20	–2.11	65.70	43.54
series II	BAD-1	10^–4^	–11.5	–1.86	–6.52	65.11	43.86
	BND-1		–10.6	–4.92	–5.79	68.36	46.65
	BND-2		–10.2	–3.22	–4.95	62.02	41.25
	BCD-1		–12.5	–2.74	–6.39	65.63	45.47

a The electrical characteristics were recorded using an Ossila Xtralien X200 sourcemeter, which provides a voltage measurement uncertainty of ±10 mV and a current uncertainty of ±10 nA.

No major deviation in *V*_OC_ is observed for the solutions of BND-1, BND-2, BCD-1, and BAD-1; however, all dyes resulted in a larger maximum *V*_OC_, and even a minimum *V*_OC_ compared to that of the bare a-Si cell. The BAD-1 dye resulted in the largest maximum *V*_OC_ value of these dyes at 1.49 V.

The *J*_SC_ measurements reported had, in general, stable values throughout the illumination cycle for all dyes and concentrations measured, although the magnitude of the values differs between the dyes tested (Figure 3 and Table 3). For the bare a-Si cell, an initial *J*_SC_ value of 22.1 A cm^–2^ was measured. Its value was slightly decreased to 21.9 A cm^–2^ when the cell reached its maximum temperature of ∼84 °C, as expected. The inclusion of the base solvent did result in an increase in *J*_SC_. The initial measurement of *J*_SC_ for the solvent indicated a value of 25.8 A cm^–2^ and eventually it rose to 26.0 A cm^–2^ when the cell reached ∼74 °C.

Dyes at 10^–3^ M concentration were found to be the least performers in terms of *J*_SC_ values compared to the bare a-Si cell. From them, the least efficient was PhEt_2_Me_4_BDP, which exhibited a maximum *J*_SC_ value of 6.30 A cm^–2^, followed closely by Me_5_BDP at 6.69 A cm^–2^. From this dye concentration, PhMe_4_BDP performed the best with a maximum *J*_SC_ value of 17.20 A cm^–2^.

Better performances for series I dyes were obtained at 10^–4^ M concentration. Even the least efficient dye at this concentration was able to outperform the best dye at 10^–3^ M concentration, with PhEt_2_Me_4_BDP providing a maximum *J*_SC_ value of 17.70 A cm^–2^. For the Me_5_BDP dye, the *J*_SC_ values varied (its maximum recorded *J*_SC_ value was 22.3 A cm^–2^, which then quickly dropped to 21.0 A cm^–2^ after 5 min of illumination (corresponding to an increase in *T*_cell_ of 5.58 °C), but even in these conditions, Me_5_BDP produced the highest *J*_SC_ value obtained for series I dyes.

The dyes in series II (Figure 4f and Tables 3 and 4) displayed more consistent *J*_SC_ recorded values throughout the entire illumination period and subsequent rise in *T*_cell_. BCD-1 dye was found to have the largest maximum *J*_SC_ value of all of the dyes tested (except for the pure solvent) at 23.6 A cm^–2^ and displayed consistency with an average *J*_SC_ value of 23.48 ± 0.11 A cm^–2^. Only BND-2 led to a reduction in *J*_SC_ values compared with the bare a-Si cell.

FF calculations resulted in no significant deviations throughout the illumination cycle for the dyes tested so far. The bare a-Si cell displayed a stable FF value of 27.42 ± 0.16% throughout the illumination cycle, with the base solvent resulting in an average of 24.08 ± 0.22%. All samples presented in Figure 4g provided a FF larger than that of the bare a-Si cell and produced the largest FFs observed in this study. PhEt_2_Me_4_BDP produced the largest FF at 47.87 ± 0.67%, while the other dyes lead to FFs ≥ 3% compared to the bare a-Si cell (Tables 3 and 4).

At the concentration of 10^–4^ M, dyes were unable to produce FFs of above 31%, yet still all outperformed the bare a-Si cell. None of the series II dyes were capable of outperforming the FF determined for the bare a-Si cell (Figure 3 and Tables 3 and 4).

**Figure 5:**
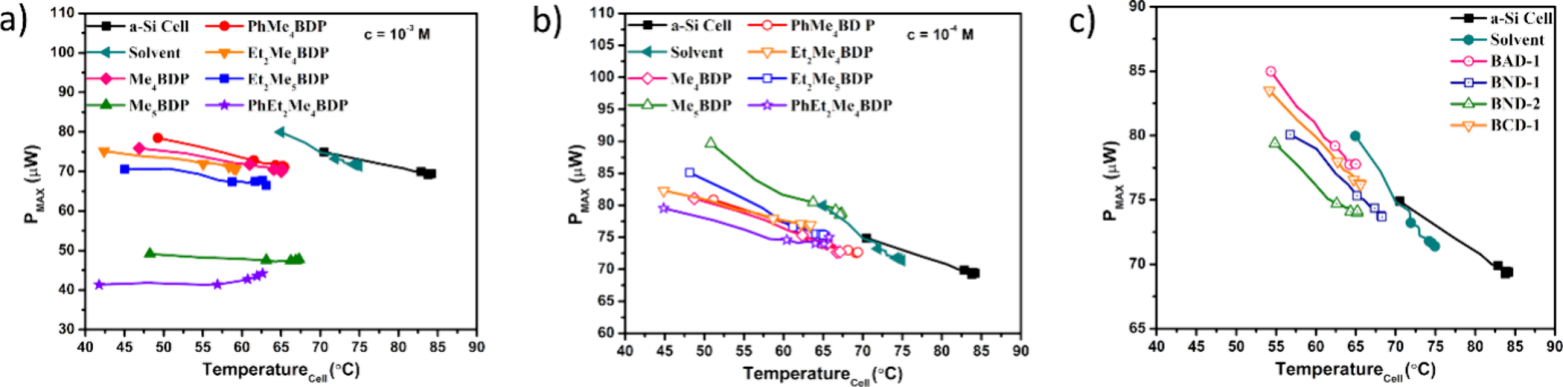
Measured *P*_max_ output for series I at 10^–3^ M concentration (a), series I at 10^–4^ M concentration (b), and series II dyes (c) throughout the illumination process of the solar cell.

The maximum power (*P*_max_) output from the solar cell when coupled with various working fluids is presented in Figure 5. The bare a-Si cell was found to produce a *P*_max_ of 74.89 μW, yet suffered a large degree of degradation over the illumination period, experiencing a rate of decline of 4.1 μW °C^–1^ (Table 4). Inclusion of the pristine solvent did offer an increase to the *P*_max_ value producing 79.95 μW, indicating that the inclusion of such a fluid would offer a potential gain in *P*_max_ values. *P*_max_ along with their decreasing rates can be found in Tables 3 and 4. A notable result for these dyes is that, throughout the illumination period, there was only a small difference in their *P*_max_ and *P*_min_ values. Dyes at 10^–4^ M concentration (Figure 5b) produced a larger *P*_max_ than that of the bare a-Si cell. Me_5_BDP produced the largest *P*_max_ value from all of the dyes tested, with a *P*_max_ value of 89.67 μW.

Series II dyes (Figure 5c) were also capable of overcoming the performances of the bare a-Si cell and those induced by the pure solvent. The largest recorded *P*_max_ is that of BAD-1, which was found to produce a *P*_max_ of 84.98 μW. BND-2 led to 79.39 μW which is below the *P*_max_ value of the pure solvent. However, the *P*_min_ recorded for the BND-2 dye is at lower operation temperatures (∼65 °C), which indicates that it provides a far more stable PV operation and electrical output than the base solvent.

### Energy Conversion Pathways and Merit Function Calculations

**Figure 6:**
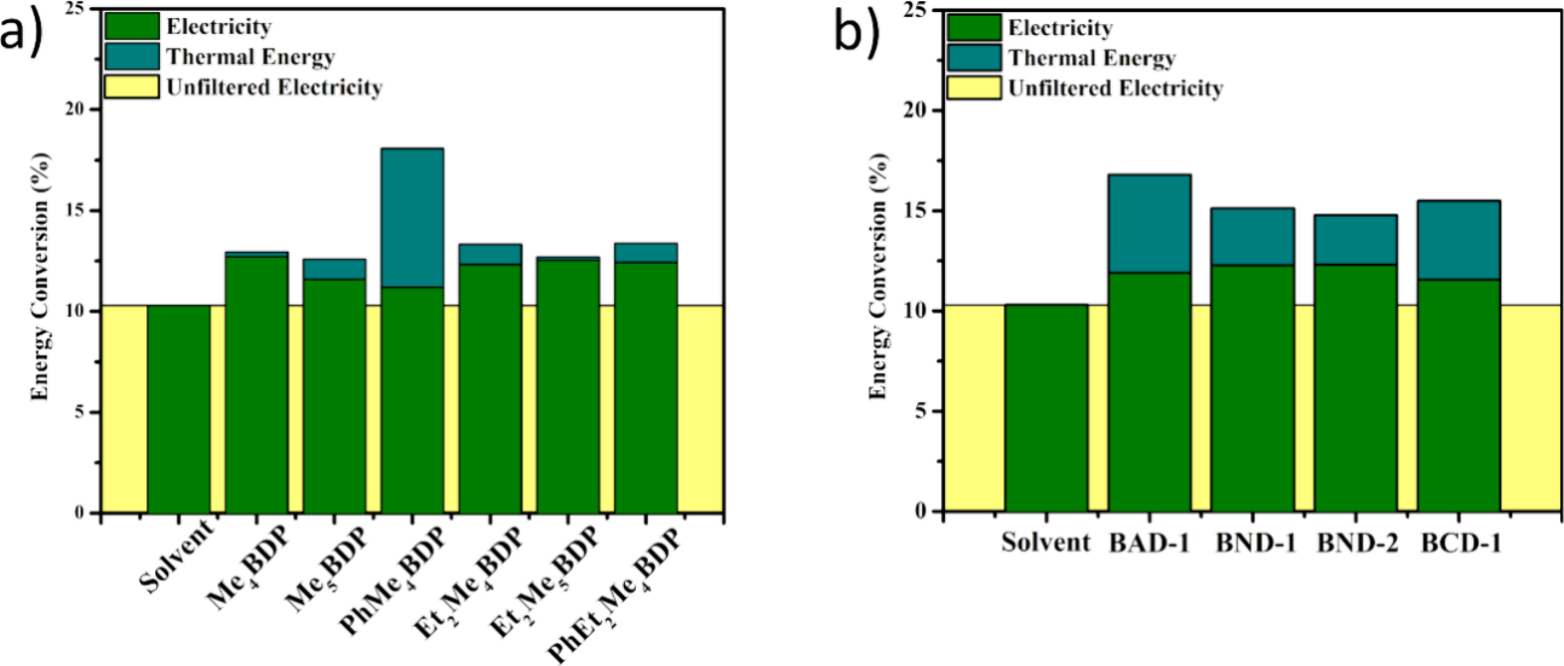
Solar energy conversion pathways of the PVT systems for series I (a) and series II (b) dyes. The energy generation pathways such as thermal (cyan) and electrical (green) are presented here, as well as the unfiltered electrical conversion (yellow), which represents the electrical energy generation from the bare a-Si cell with no additional PVT fluid being present.

While the electrical and thermal evaluations offer direct individual insights into the performance of the a-Si cell under various conditions, the solar energy conversion efficiencies for both the electrical and thermal pathways should be evaluated through a merit function calculation in order to understand the overall performance of the PVT systems (Figure 6 and Table 5). This analysis allows a detailed view of the energy generation pathways for the hybrid PVT system.

**Table 5:** Electrical and Thermal Energy Conversion Pathways for Each Compound, along with the Calculated Merit Function Value

series	compound	electrical energy generation (%)	thermal energy generation (%)	merit function
	decalin	10.30	0.00	0.33
series I	Me_4_BDP	12.71	0.24	0.24
	Me_5_BDP	11.59	1.00	0.46
	PhMe_4_BDP	11.19	6.87	1.03
	Et_2_Me_4_BDP	12.32	1.01	0.50
	Et_2_Me_5_BDP	12.54	0.15	0.42
	PhEt_2_Me_4_BDP	12.43	0.95	0.49
series II	BAD-1	11.91	4.89	0.86
	BND-1	12.28	2.84	0.67
	BND-2	12.31	2.47	0.64
	BCD-1	11.56	3.94	0.76

The solar energy conversion of the bare a-Si cell is, in fact, its electrical output and is represented by “Unfiltered Electricity” in Figure 6. Thus, bare a-Si used in this study had a solar energy conversion efficiency of 10.30%, and it represents only electrical energy generation because there are no means to recover any thermal component. Therefore, the high operational temperature of the bare a-Si cell will be detrimental to its efficiency and its lifetime.

The use of the pure solvent (i.e., not containing any dye) resulted in an electrical conversion efficiency that matches that of unfiltered electricity generated with a conversion efficiency of 10.30%. This result is expected because the solvent does not absorb light in the visible region; hence, its electrical conversion should be akin to that of the unfiltered a-Si cell. Lack of light absorption also accounts for the thermal energy generation pathway providing 0% thermal energy because no energy is absorbed by the fluid in this state.

Before further examination of each of the dyes tested, it should be mentioned that all dyes outperformed the solvent in terms of the electrical conversion efficiency alone (Figure 6). Even without the need of providing additional thermal energy generation, each dye provides a direct benefit to the system and is viable as a hybrid PVT fluid. Thus, even though the energy output will be similar to that provided by the bare a-Si cell, the fact that the operation of the PV is at lower temperature will lead to longer lifetime.

For the dyes seen in Figure 6a, as previously mentioned, all are capable of providing an increase in the total solar energy conversion through electrical and thermal pathways. The effect of this thermal energy generation is small for Me_4_BDP and Me_5_Et_2_BDP, which were shown to convert 0.24% and 0.15% of thermal energy, respectively. However, they both provided an increase to the electrical energy generation of 2.41% and 2.24%, respectively, compared to the bare a-Si electricity conversion. The 2.41% of electrical energy conversion observed for the Me_4_BDP dye was, in fact, the largest gain in electrical energy conversion across any dye represented in Figure 6. This gain implies that more photons with wavelengths within the PV spectral window are now reaching the PV. While the gain in electrical energy conversion is a positive result, the ability to also generate thermal energy is an added benefit to the hybrid PVT system. As such, the dyes tested were capable of providing this thermal energy generation, albeit to a minor degree, across the whole series. Of the five dyes tested in this category, only two were capable of providing more than 1.00% thermal energy generation. The PhMe_4_BDP dye offers the largest thermal energy generation production across all dyes. This dye provides a thermal energy generation of 6.87%, much larger than that of any other dye tested. However, the tradeoff of this dye is that the electrical energy generated was only 11.19%. The total energy generation for this dye is 18.07% compared to 10.30% for the unfiltered electricity, which further highlights the potential of this dye as a viable PVT fluid.

The series II dyes shown in Figure 6b were also found to provide positive results, particularly in terms of the thermal energy generation. Except for PhMe_4_BDP, all dyes in series II were able to provide more than double the rate of thermal energy generation compared with series I. BND-2 provided the smallest thermal energy conversion value at 2.47%. The BAD-1 dye provided the largest thermal energy generation pathway for this series of dyes by producing 4.89% thermal energy generation. This dye was also capable of increasing electrical energy generation by providing an increase of 1.60% compared to unfiltered electrical energy.

Primary methods of evaluating the potential of a PVT fluid can be achieved through an analysis of its merit function value.^51^ The merit function value (Table 5) will offer insights into the potential for a fluid to efficiently convert energy. The pure solvent produced a merit function value of 0.33 and hence determines the baseline comparison for each subsequent dye. A higher value will determine a higher viability as a successful candidate as a PVT fluid. For the series I dyes presented in Figure 6a, all samples produced a higher merit function value than that of the pure solvent. This is an expected result due to the greater potential for these dyes to increase the electrical energy generation while also providing a small additional thermal energy generation increase. The PhMe_4_BDP dye produced a merit function value of 1.03, which is the highest merit function value for both series of dyes. This result is due to the significant potential for thermal energy generation. Series II dyes produced consistent merit function values between 0.64 (BND-2) and 0.86 (BAD-1). The consistency of the calculated merit function of the series II dyes compared to the series I dyes is due to their more consistent electrical energy generation increases as well as their good thermal energy generation. While the electrical energy generation of the series II dyes is not as large as that of the series I, there is less variation in their thermal energy pathways, which results in this benefit.

## Conclusion

In this study, two series of dyes were synthesized and utilized to address the spectral mismatch between the incoming spectrum and PV cells through a LDS process. Typically, in a PVT system, the enhancement in solar energy conversion is based on a decrease in electrical energy generation due to the light absorption by the liquid, which is compensated by a significant increase in thermal energy generation. However, this research demonstrates that enhancement of PVT solar energy conversion can be achieved without any detrimental effect on electrical energy generation. On the contrary, it shows that enhancement can be realized by increasing both electrical and thermal energy generation. This indicates that more photons within the PV spectral window reach the PV cells. Additionally, the use of the liquid helps to reduce the operating temperature of the PV cells, potentially contributing to an extension of the PV module’s lifetime. These findings highlight a novel and effective approach to improving the performance and durability of PVT systems through the incorporation of luminescent dyes, offering significant potential for advancements in solar energy technology.

The obtained data indicate a strong correlation between the overall effect of the working liquid on the cell performance and both the concentration of the dye and its fluorescence quantum yield. Increasing the concentration of the dye reduced the thermal load on the bare a-Si cell. For example, for series I, a concentration of 10^–3^ M resulted in a *T*_cell_ reduction of approximately 25 °C, while a concentration of 10^–4^ M provided a reduction of up to 21 °C. However, higher dye concentrations lead to a reduction in the electrical performance due to the diminished photon flux reaching the cell, as evidenced by the short-circuit current density data. Dyes at a concentration of 10^–4^ M performed better than those at 10^–3^ M, with Me_5_BDP achieving the highest *J*_SC_ of 22.30 A cm^–2^.

Among the series I dyes, PhMe_4_BDP stood out with the highest thermal energy generation of 6.87%, resulting in an enhancement of the total solar energy conversion by 18.07%, despite having a lower electrical gain than the other series I dyes. This dye possesses the lowest fluorescence quantum yield (0.4213) in the series due to enhanced nonradiative decays of the excited state in this molecule. Thus, despite the expected poor performance as an LDS material, the overall performance of this compound is significant due to its better ability to dissipate heat compared to other molecules.

Interestingly, all dyes in series II, which include structures based on the same 1,3,5,7-tetramethylBODIPY scaffold as that in PhMe_4_BDP, demonstrated good thermal and electrical performances. However, none of them could outperform PhMe_4_BDP. This is likely due to the higher emission quantum yields in this series, which reduce the dye’s ability for thermal energy conversion. This result is surprising and demonstrates that the balance between radiative and nonradiative transition rates in the dye can significantly affect energy gains in the PVT system. Dyes with weaker fluorescence can potentially provide higher overall energy gains due to enhanced thermal energy conversion.

In summary, the enhancement obtained in solar energy conversion indicates that the inclusion of BODIPY dyes can lead to significant energy gains in PVT systems. This study demonstrated that the incorporation of these dyes can improve both the thermal and electrical components of solar energy conversion, highlighting their potential to mitigate thermal loads and extend the lifespan of PV modules. Among the studied dyes, those with moderate fluorescence quantum yields showed the most promise, suggesting that an optimal balance between radiative and nonradiative transitions is crucial for maximizing the overall efficiency.

However, further studies are needed to identify dyes with optimal fluorescence parameters that will provide a balance between thermal and electrical components in the overall efficiency gain achieved using PVT liquids. This includes exploring a wider range of dye concentrations and molecular structures to refine the balance among photon absorption, emission, and heat dissipation. The insights gained from this research pave the way for future developments in PVT technology with the aim of more efficient and durable solar energy systems.

## Supporting Information Available

^1^H and ^13^C NMR, HRMS, UV absorption, and fluorescence spectra, fluorescence quantum yields in different solvents, and a scheme of the experimental setup for PVT experiments (PDF)
