# Supplementary material for: Enhancing Hybrid Photovoltaic–Thermal System Efficiency with Boron Dipyrromethene Dyes
Source: ACS Appl Opt Mater. 2024 Sep 4;2(9):1985–98. doi: 10.1021/acsaom.4c00309 (PMC11443531; doi:10.1021/acsaom.4c00309)
Supplement: Supplementary file 1 — ot4c00309_si_001.pdf [file ot4c00309_si_001.pdf]

Supporting Information for

Enhancing Hybrid Photovoltaic-Thermal System Efficiency with BODIPY Dyes

Kenneth Coldrick,[a,b] Craig Newman,[c] John Doran, [b] George Amarandei,[a,b]* and Mikhail A. Filatov[c]*

[a] School of Physics, Clinical & Optometric Sciences, Technological University Dublin, City Campus, Grangegorman Lower, D07 ADY7 Dublin, Ireland. E-mail: george.amarandei@tudublin.ie

[b] The Group of Applied Physics, Technological University Dublin, City Campus, Grangegorman Lower, D07 ADY7 Dublin, Ireland

[c] School of Chemical and Biopharmaceutical Sciences, Technological University Dublin, City Campus, Grangegorman Lower, Dublin 7, Ireland. E-mail: mikhail.filatov@tudublin.ie

Table of Contents

1. NMR Spectra 2

2. Mass Spectra 5

3. UV-Vis Absorption and Photoluminescence Spectra 6

4. PVT Experiments 8

5. References 9

1. NMR Spectra

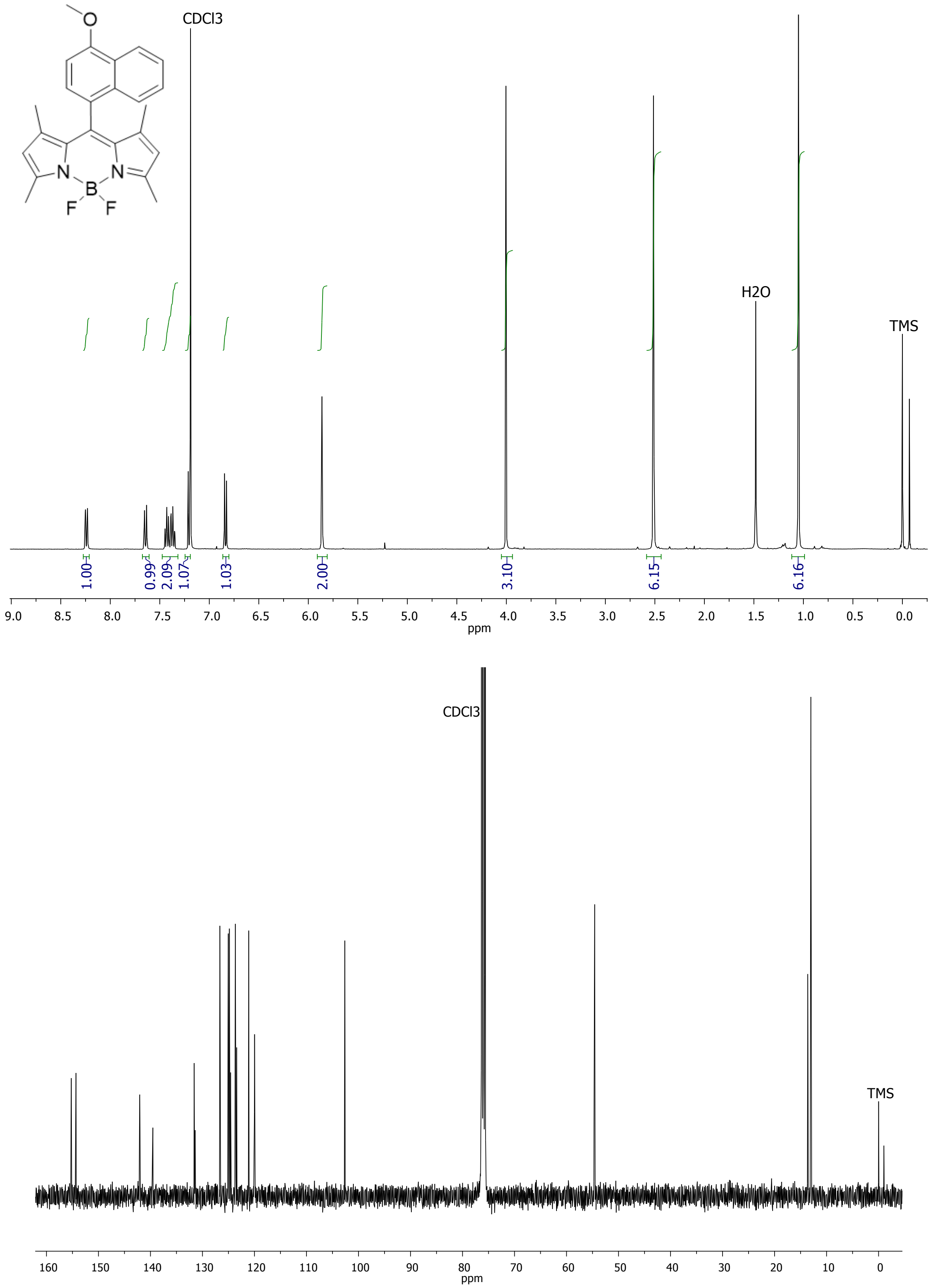

Figure S1. ^{1}H and ^{13}C NMR spectra of compound **BND-1.**

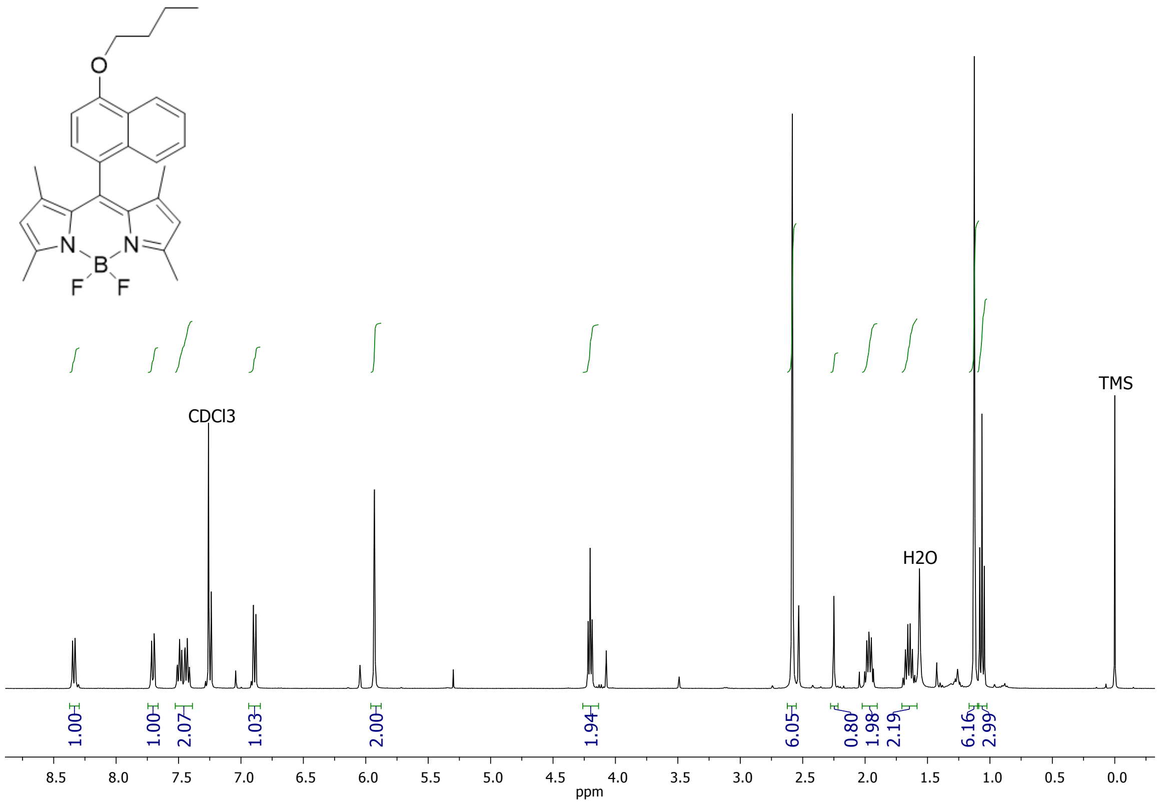

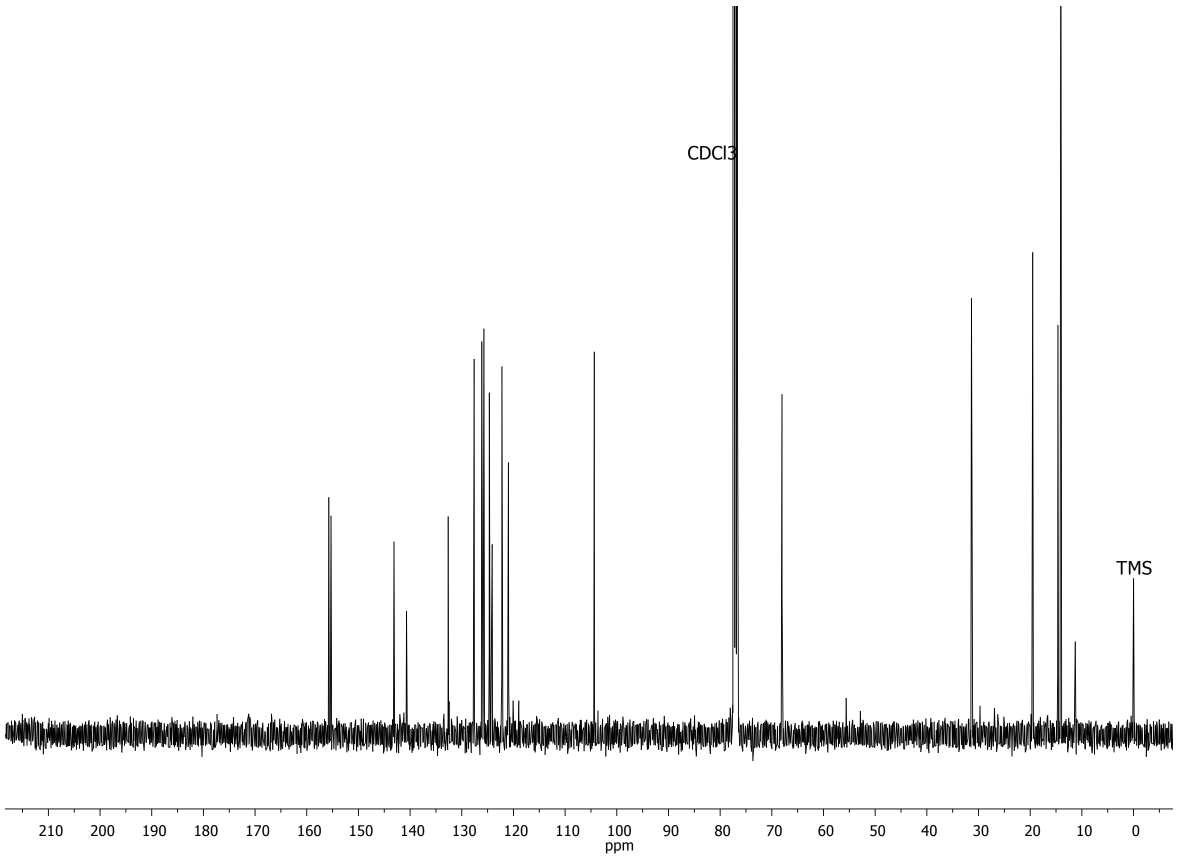

Figure S2. ^{1}H and ^{13}C NMR spectra of compound **BND-2.**

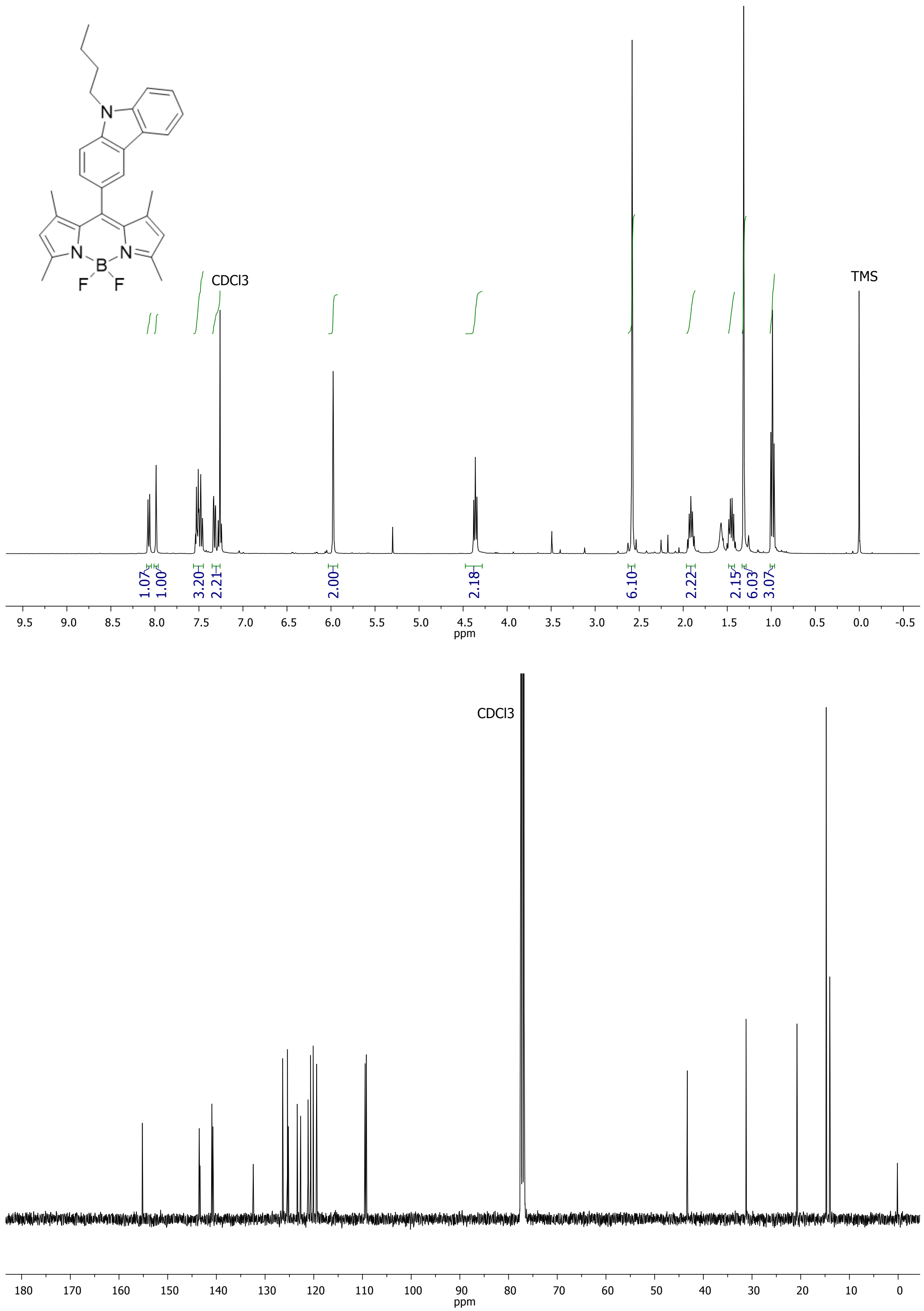

Figure S3. ^{1}H and ^{13}C NMR spectra of compound **BCD-1.**

2. Mass Spectra

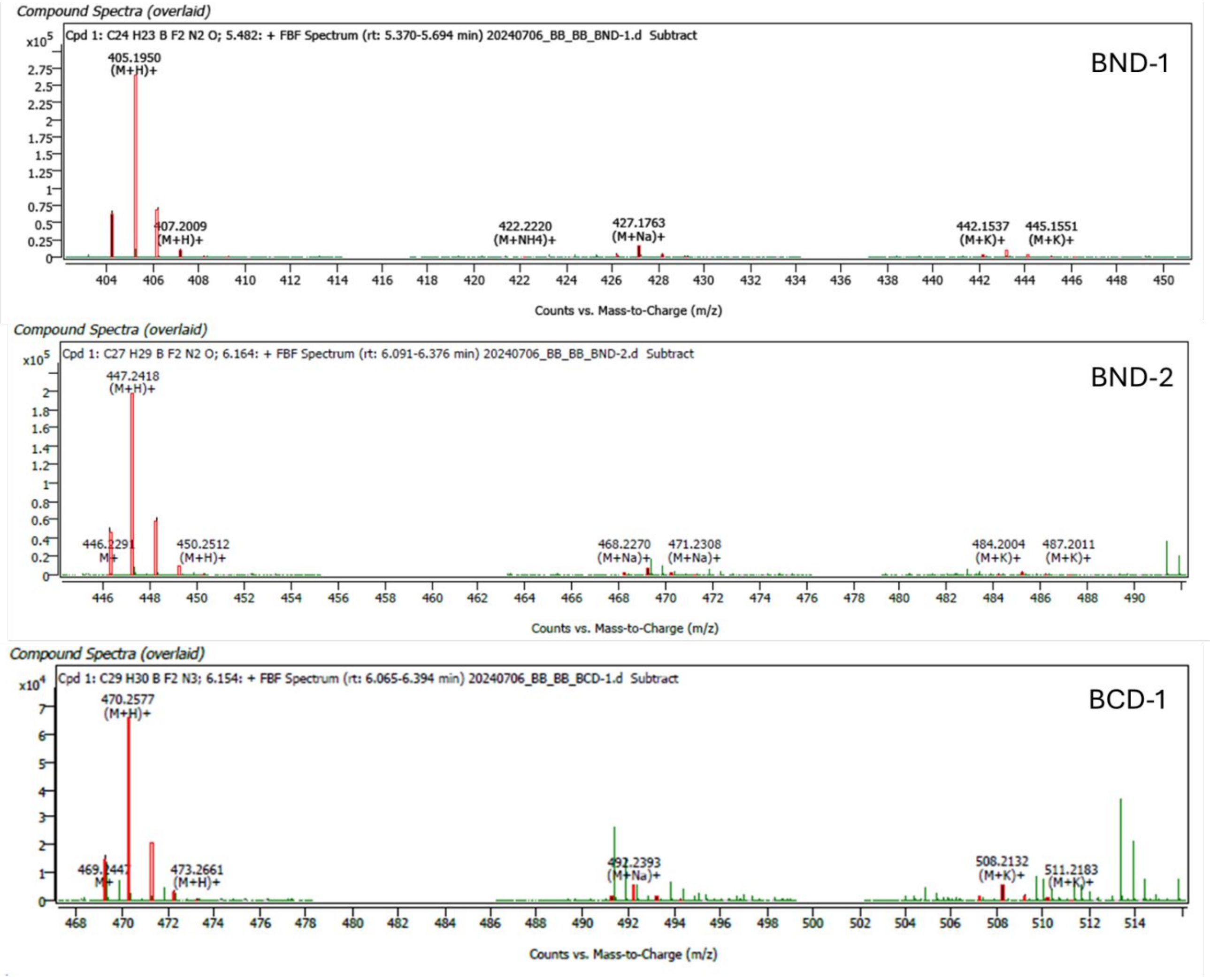

Figure S4. QTOF HRMS spectra of the prepared compounds.

3. UV-Vis Absorption and Photoluminescence Spectra

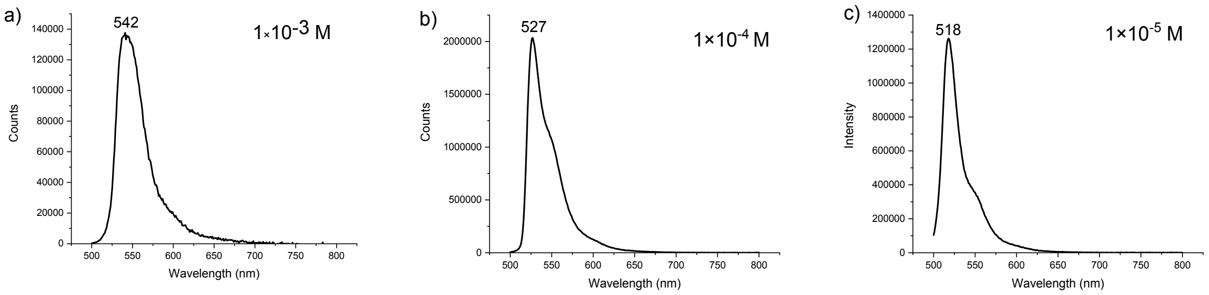

Figure S5. Emission spectra of BAD-1 recorded at different concentrations in toluene (λ_{exc} = 470 nm).

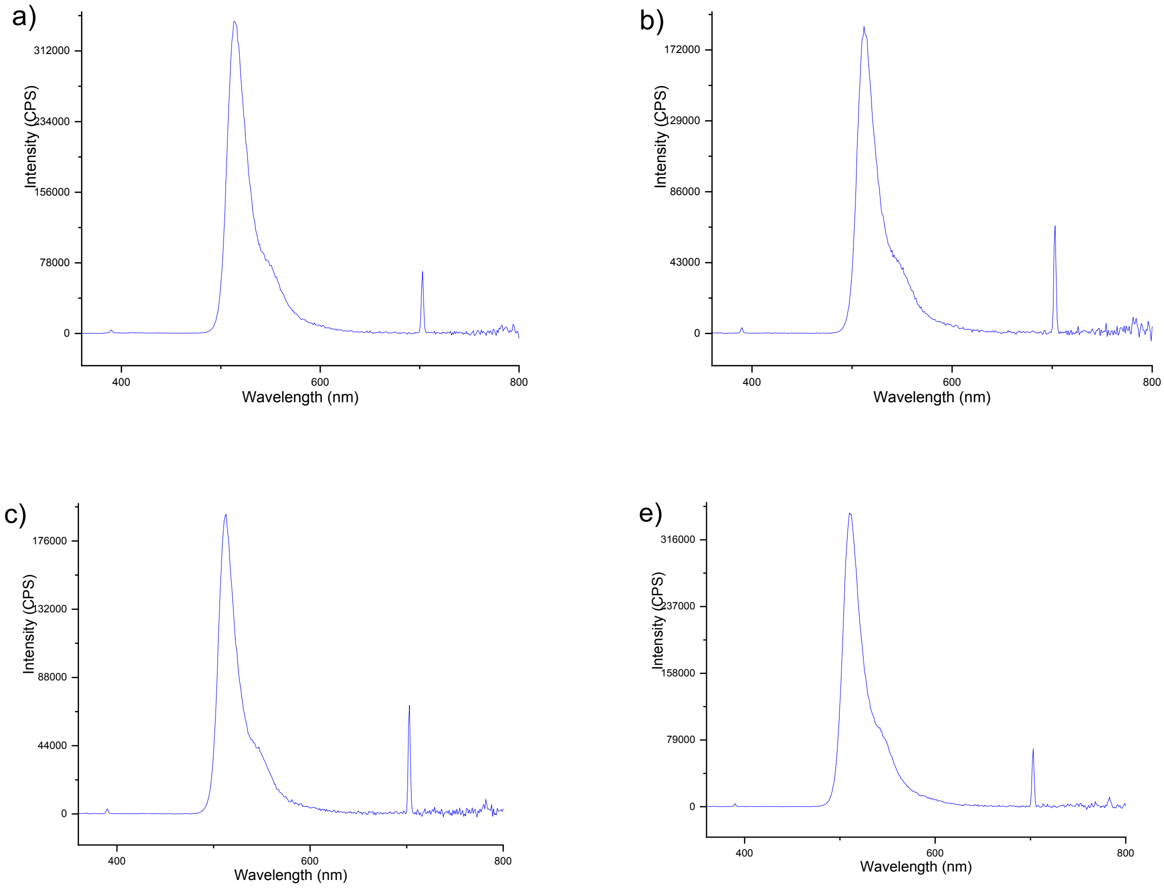

Figure S6. Emission spectra of BAD-1, BND-1, BND-2 and BCD-1 under excitation at 350 nm in cyclohexane. Peak at 700 nm corresponds to the second harmonic generation.

Table S1. Absolute fluorescence quantum yields of BAD-1 in cyclohexane at different concentration.

Compound	Concentration (M)	Absolute PLQY (%)
BAD-1	1×10^{-3}	72.5
	1×10^{-4}	74.1
	1×10^{-5}	75.9

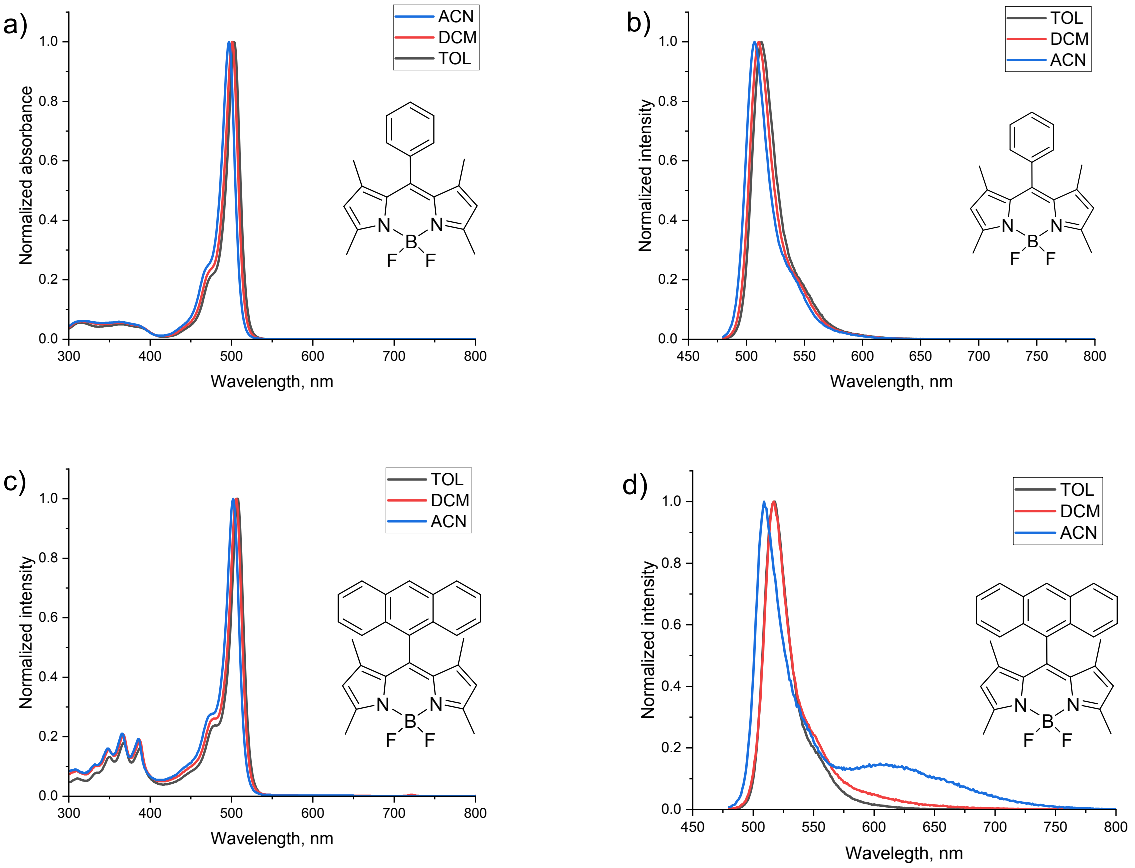

Figure S7. Absorption and emission spectra of $PhMe_4BDP$ (a,b) and BAD-1 (c,d) in different solvents. Excitation wavelength: 470 nm.

Table S2. Fluorescence quantum yields of $PhMe_4BDP$ and BAD-1 in different solvents.

Compound	Solvent	λ_{em} (nm)	Φ_{em}
$PhMe_4BDP$	toluene	503	0.64
	DCM	501	0.553
	CH_3CN	497	0.466
BAD-1	toluene	521	0.92
	DCM	517	0.072
	CH_3CN	512, 621	0.006

4. PVT Experiments

The schematic of the experimental setup[1] used to investigate the PVT system is presented in Figure S8. As a means of mimicking the AM1.5G solar spectrum of normal incidence on a typical PVT device, an Abet 10500 Solar Simulator lamp was positioned at a working distance of 10 cm in relation to the fluid under investigation. The Abet 10500 Solar Simulator is a Class A characterised solar lamp with an AM1.5G air mass filter provided by a DC Xenon Arc Lamp. To evaluate the intensity of the light, which was produced by this spectral lamp, a Kipp and Zonen SP Lite2 pyranometer was connected to a standard multimeter and the distance of the working fluid was adjusted to read an equivalent irradiance off 1000 Wm^{-2} incident on the pyranometer. A screw cap quartz cuvette (spectral range 190-2500 nm, pathlength 10x10 mm, chamber volume 3500 μL, eCuvettes, Sichuan, China) was filled with the working fluid and placed in a custom 3D printed holder which positioned it within the optical path of the Abet 10500 solar simulator.

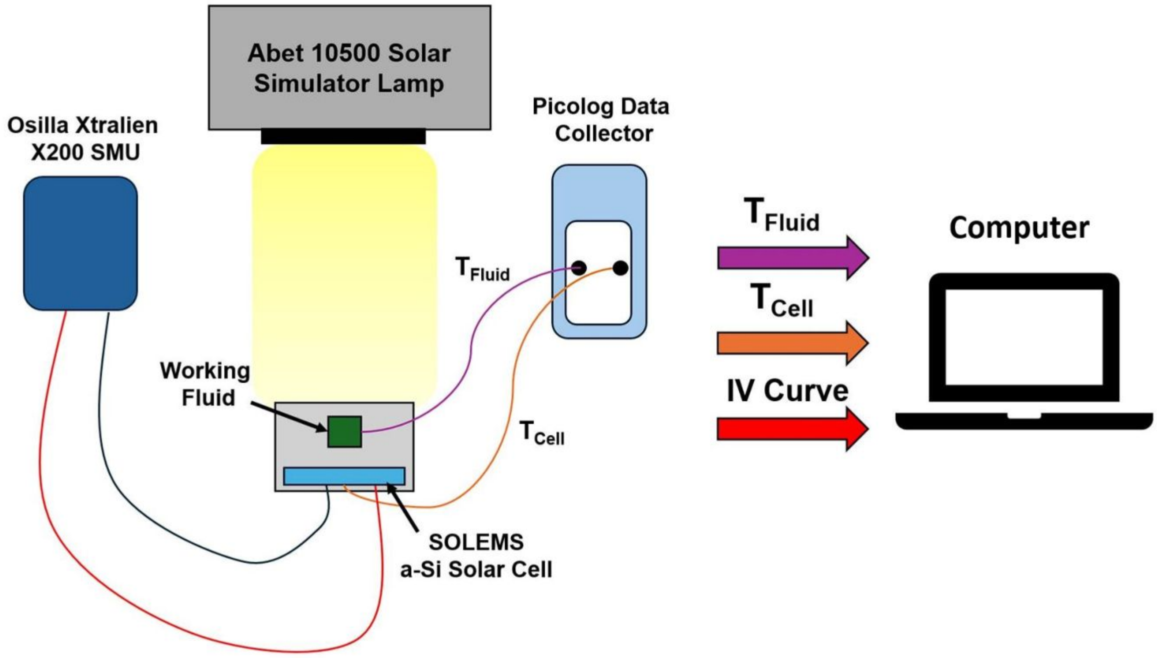

Figure S8. Top view schematic of the experimental photovoltaic thermal (PVT) setup used in this study. An AM1.5G solar spectrum is produced by the Abet 10500 solar simulator which provides normal incident light onto the sample holder containing the working fluid and the a-Si PV cell. The "TC-08 logger PicoLog" and "Ossila Source Measure Unit" with their appropriate software packages were used for gathering the data on temperature and electrical characteristics, respectively.

5. References

[1] K. Coldrick, J. Walshe, S.J. McCormack, J. Doran, G. Amarandei, *Energies*, **2023**, *16*, 6294.
